# Detecting Migraine in Patients with Mild Traumatic Brain Injury Using Three Different Headache Measures

**DOI:** 10.1155/2015/693925

**Published:** 2015-05-27

**Authors:** Kirsten Anderson, Simon Tinawi, Julie Lamoureux, Mitra Feyz, Elaine de Guise

**Affiliations:** ^1^Psychology Department, University of Montreal, Pavillon Marie-Victorin, CP 6128, Succursale Centre Ville, Montreal, QC, Canada H3C 3J7; ^2^Physical Medicine and Rehabilitation Service, McGill University Health Centre, 1650 Cedar Avenue, Montreal, QC, Canada H3G 1A4; ^3^Social and Preventive Medicine Department, University of Montreal, 7101 Avenue du Parc, Montreal, QC, Canada H3N 1X7; ^4^Traumatic Brain Injury Program, McGill University Health Centre, 1650 Cedar Avenue, Montreal, QC, Canada H3G 1A4; ^5^Neurology and Neurosurgery Department, McGill University Health Centre, Montreal, QC, Canada H3G 1A4

## Abstract

Posttraumatic migraine may represent an important subtype of headache among the traumatic brain injury (TBI) population and is associated with increased recovery times. However, it is underdiagnosed in patients with mild traumatic brain injury (mTBI). This study examined the effectiveness of the self-administered Nine-Item Screener (Nine-Item Screener-SA), the Headache Impact Test- 6 (HIT-6), the 3-Item Migraine Screener, and the Rivermead Post-Concussion Questionnaire (RPQ) at discriminating between mTBI patients with (*n* = 23) and without (*n* = 20) migraines. The Nine-Item Screener demonstrated significant differences between migraine patients with and without migraine on nearly every question, especially on Question 9 (disability), sensitivity: 0.95 and specificity: 0.65 (95% CI, 0.64–0.90). The HIT-6 demonstrated significant differences between migraine and no-migraine patients on disability and pain severity, with disability having a sensitivity of 0.70 and specificity of 0.75 (95% CI, 0.54–0.83). Only Question 3 of the 3-Item ID Migraine Screener (photosensitivity) showed significant differences between migraine and no-migraine patients, sensitivity: 0.84 and specificity: 0.55 (CI, 0.52–0.82). The RPQ did not reveal greater symptoms in migraine patients compared with those without. Among headache measures, the Nine-Item Screener-SA best differentiated between mTBI patients with and without migraine. Disability may best identify migraine sufferers among the TBI population.

## 1. Introduction

Posttraumatic headaches (PTH) are common in the TBI population, with a prevalence ranging from 30 to 90% depending on the type of measurement instrument administered to patients [1]. Posttraumatic headaches may persist long after the TBI, with 18% to 22% of PTH sufferers still experiencing symptoms one year later [2]. Approximately 75% of the 1.7 million patients with traumatic head injuries in the United States (annual) are classified as mild [3]. Posttraumatic headaches in this population may be the major cause of persistent disability. The frequency of different headache types after TBI can be quite variable in different studies. An important reason underlying this variability may be differences in the measurement instruments used to classify them. Several widely used and well-validated tests have been designed to measure the specific characteristics of headache and headache-related disability, including the Headache Disorders, 2nd Edition (ICHD-II), the Nine-Item Screener [4], the Headache Impact Test (HIT-6) [5], and the Three-Item ID Migraine Screener [6]. Few studies have examined the efficacy of these tests in distinguishing between mild TBI patients with and without migraines.

Classification of PTH types is useful for both research and treatment. Posttraumatic headaches are classified as secondary headaches. However, they have no clear defining features that distinguish them from primary headaches other than their close temporal relationship with the TBI. As such, primary headache questionnaires that distinguish headache types according to duration, frequency, and character of pain are often applied to PTH [7]. A significant number of posttraumatic headaches fall into the category of the migraine phenotype, when using the International Classification of Headache Disorders, Edition 2 (ICHD-II) [7, 8]. Posttraumatic migraines can lead to significant disability, even following mild TBI [9].

Using the primary headache criteria, migraine headaches are defined as moderate to severe headaches that may be accompanied by systemic problems such as nausea and vomiting, pain worsening with activity, and photosensitivity [3]. Migraines can also disturb cognitive function, vestibular function, emotional state, and social interactions. Thus, they may be debilitating and impede patients' recovery from TBI [3].

An accurate diagnosis of posttraumatic migraines is essential for proper treatment. The management of headache necessitates the use of analgesics such as acetaminophen and ibuprofen, whereas migraine sufferers are often prescribed antinausea medications and preventive or abortive treatments, such as triptans [8, 10]. Unfortunately, a substantial proportion of the mild TBI population does not receive adequate management for their migraines. This may be due to inadequate classification [8] or failure to seek medical attention. In spite of the known diversity of primary headache types, 70% of patients with mild TBIs rely solely on over-the-counter nonspecific medications such as acetaminophen and nonsteroidal anti-inflammatories to treat their headache symptoms [8]. Amongst posttraumatic migraine sufferers, only 26% experience symptom relief [8]. Preventive treatment can lead to attacks that are less severe and shorter in duration [10]. Furthermore, excessive use of over-the-counter analgesics may provoke medication-overuse headache [8, 11, 12]. This can lead to further management challenges and poorer functional outcome [12].

Posttraumatic migraine sufferers are reported to have longer recovery times from mild TBI than other PTH patients [9, 13, 14]. In one study, patients suffering from posttraumatic migraines demonstrated a 7.3 times greater risk for extended recovery time than nonmigraine TBI patients [9]. Disability is a key factor in planning level of care based on the patient's work and family obligations.

The goal of the present study is to determine which of the commonly used headache questionnaires best detects migraines in a population of mild TBI patients. We compared the sensitivity and specificity of the Nine-Item Screener, the HIT-6, and the Three-Item ID Migraine Screener at detecting and distinguishing patients with migraines from those without migraines in a mild TBI population. By focusing on these commonly used, well-validated tests, this work aims to better characterize the usefulness of different headache inventories in the TBI population and may serve as an important basis for diagnostic classification for research and treatment purposes.

## 2. Materials and Method

### 2.1. Participants

Forty-three patients with mild traumatic brain injury were seen at the Outpatient Clinic at the McGill University Health Centre-Montreal General Hospital (MUHC-MGH) between September 1, 2012, and August 1, 2013, and consecutively enrolled in the present study. The patients had recently sustained a head trauma and were diagnosed with an mTBI by a physician who used WHO Task Force Criteria [15], which includes at least one of the following symptoms: posttraumatic amnesia of less than 24 hours, loss of consciousness of up to 30 minutes, seizure, focal signs, disorientation, or scans demonstrating intracranial lesions that do not require surgery. The etiology of injury was varied (Table 1). This study was approved by the MUHC-MGH Research Ethics Board.

### 2.2. Pretrauma Sociodemographic Characteristics, Clinical Variables, and Accident Variables

Data was collected from the TBI program database maintained at the MUHC-MGH. Accident etiology and duration of posttraumatic amnesia were examined, as well as gender, and history of headaches and migraines (Table 1). Migraine patients included those with (*n* = 3) and without (*n* = 20) aura.

### 2.3. Procedure

Self-report questionnaires were administered to patients before the medical evaluation, an average of 43.24 days (SD = ±29.96) after TBI. Next, a physician specializing in rehabilitative medicine performed a migraine assessment based on 2004 ICHD-II criteria for migraine [16], the HIT-6, family history of headache, cervical sprain, childhood motion sickness, and food intolerance. He then conducted a neurological examination.

### 2.4. Measures

#### 2.4.1. Headache Disorders, 2nd Edition (ICHD-II): Nine-Item Screener [4]

In the present study, this widely accepted physician-administered questionnaire was self-administered by patients. It is based on IHS criteria and consists of nine yes or no questions that serve to identify and categorize headaches into a hierarchical system. The questions help characterize pain and aura. They also help identify nausea, light and sound sensitivity, and functional impairment. The sum of positive answers is calculated to obtain a total score.

#### 2.4.2. The Headache Impact Test (HIT-6) [5]

This self-report questionnaire can be used as both a screening tool and a way of measuring changes in headache impact. It consists of six questions that are designed to measure the impact that headaches have on the patient's normal function in social situations and at work, home, and school. Items are on a rating scale of 1–5 that includes never, rarely, sometimes, very often, and always. Scores of 50 or higher indicate that the patient requires medical attention.

#### 2.4.3. Three-Item ID Migraine Screener [4]

This self-report questionnaire consists of three yes or no items and can be used as a screening tool in a primary care setting to identify headaches, nausea, light sensitivity, and functional impairment.

#### 2.4.4. The Rivermead Post-Concussion Symptoms Questionnaire [6]

This self-report questionnaire measures the severity of cognitive, emotional and somatic symptoms in TBI patients. It consists of 16 questions on a rating scale including 0 (not experienced at all), 1 (no more of a problem), 2 (a mild problem) 3 (a moderate problem) and 4 (a severe problem). If at least three symptoms are present at three months, the patient is considered to have Post-Concussion Syndrome [17].

## 3. Results

### 3.1. Data Integrity

Three patients did not complete the HIT-6 questionnaire. There were no values missing for the Nine-Item Screener. Two or three values were missing on the 3-Item ID Migraine Screener, depending on the question, due to incomplete information provided by patients.

### 3.2. Descriptive Statistics

Twenty of the forty-three mild TBI patients suffered migraines, according to ICHD-II classification. Ages ranged from 26 to 63 with an average of 44.8 years (SD = 18.3). Approximately half (51.2%) of the patients were female. There were no statistically significant differences between the migraine and no-migraine groups in terms of previous TBIs, cervical sprains, duration of posttraumatic amnesia, and length of stay (Table 1). Groups also did not differ significantly on psychiatric problems, education, or alcohol and drug abuse. Finally, no significant differences were found between groups on food intolerance and motion sickness.

### 3.3. Headache Test Results

#### 3.3.1. Headache Disorders, 2nd Edition (ICHD-II): Nine-Item Screener-SA [4]

**Figure 1:**
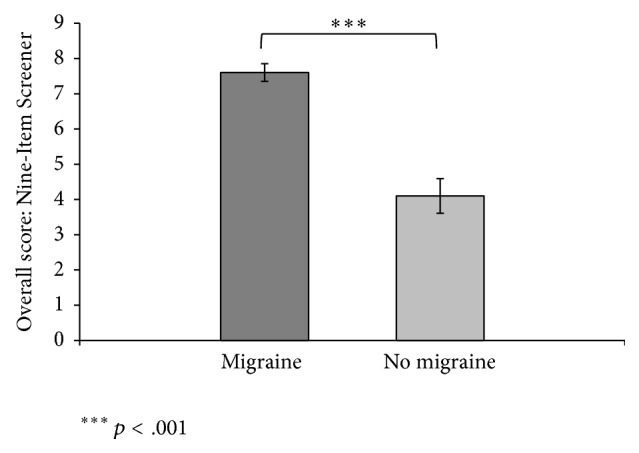
Mean values of migraine scores for migraine and no-migraine patients on the Nine-Item Screener. Standard errors of the mean are represented in the figure by the error bar attached to each column.

Chi-square tests were used to compare scores between the migraine group and the no-migraine group on each question. There was a higher frequency of migraine symptoms and lower functioning in the migraine group compared with that of the no-migraine group on all questions except number four (Table 2). To evaluate the validity of the Nine-Item Screener as a predictor of migraine headaches in the mild TBI population, sensitivity and specificity were examined. Item nine was the most predictive (Table 3). Finally, since each item of the Nine-Item Screener-SA reflects only the absence/presence of symptoms, an overall score was calculated to compare the migraine group to the no-migraine group. The mean number of symptoms in the group with migraines was 7.60 (SD = 1.60), with a median of 5 and a range of 0 to 9. The group without migraines had a mean of 4.2 (SD = 3.1), with a median of 8 and a range of 3 to 9. The distribution was not normal; therefore the nonparametric Wilcoxon rank sum test was used. Migraine patients reported significantly more symptoms than those without migraines, *z* = 3.78, *p* < .001 (Figure 1). The sensitivity of the total score was 90.0% and the specificity was 69.9%, while Question 9 had a sensitivity of 95.0% and a specificity of 65.2%, indicating that this question alone has almost as much validity as the overall score.

#### 3.3.2. The Headache Impact Test (HIT-6) [5]

The HIT-6 total score was compared in the migraine group versus the no-migraine group using a two-tailed independent samples *t*-test. There were marginally significant differences between the migraine group (*M* = 2.60; SD = 0.70) and the nonmigraine group (*M* = 2.10; SD = 1.00), *t*(38) = 3.82;  *p* = .076. Chi-square tests were then performed on specific questions to determine whether migraine symptoms and functional problems in daily life were more frequently experienced by migraine patients than the no-migraine group. Migraine patients reported a statistically significant higher frequency of symptoms than nonmigraine patients on item one, *χ* ^2^  (4, *N* = 40) = 11.47, *p* = .022. To evaluate the validity of the HIT-6 as a predictor of migraine headaches in the mTBI population, the sensitivity and specificity were evaluated. At a cut-off of “very often,” item one had a sensitivity of 70% and a specificity of 75%. Migraine patients also demonstrated a higher frequency of symptoms on the second question, *χ* ^2^  (4, *N* = 40) = 9.57, *p* = .048. Item two had a sensitivity of 60% and a specificity of 70%. There was no statistically significant difference between groups on the last four questions (Table 4).

#### 3.3.3. The 3-Item ID Migraine Screener [4]

Chi-square tests were used to compare scores between the migraine group and the no-migraine group on each question. Item 2, light sensitivity, was the only item that revealed statistically significant differences between the migraine and no-migraine groups *χ* ^2^  (1, *N* = 41) = 6.60; *p* = .010 (sensitivity = 84.2%; specificity = 54.6%). This item was also the only one with good predictive power (sensitivity = 84.2%; specificity = 54.6%; Table 5).

#### 3.3.4. The Rivermead Post-Concussion Symptoms Questionnaire (RPQ) [6]

The RPQ total score was compared between patients with and without migraine, using a two-tailed independent samples *t*-test. No statistically significant differences were found between the migraine group (*M* = 33.40; SD = 13.08) and the nonmigraine group (*M* = 32.52; SD = 17.77); *t*(38) = 0.18; *p* = .859.

## 4. Discussion

To our knowledge, previous studies have not examined the effectiveness of different headache tests at detecting migraine in a mild TBI population. Therefore, the main purpose of this study was to determine which headache tests among the Nine-Item Screener-SA, the HIT-6, and the 3-Item ID Migraine Screener would best differentiate between mild TBI patients with and without migraines. Results on the Nine-Item Screener and the HIT-6 demonstrated that migraine patients experienced more disability than nonmigraine patients. Across headache inventories, the items that best detected differences between TBI patients with and without migraines were pain severity, disability, and photosensitivity, with migraine patients reporting greater levels of each. The headache test that best differentiated between migraine patients and nonmigraine patients was the Nine-Item Screener, which demonstrated differences on nearly every item. The HIT-6 and the 3-Item ID Migraine Screener appeared to be less sensitive for this purpose. The RPQ did not demonstrate higher scores among migraine patients.

Posttraumatic migraine has been identified as an important subtype of headache among the TBI population due to the compounded cognitive and physical disability associated with this diagnosis [3]. Functional disability is also high in migraine sufferers in the general population. Indeed, one study reported that 75% of migraineurs felt they required complete bed rest and suffered severe disability during a migraine [18]. Furthermore, Kontos and colleagues found that migraine patients' scores on computerized neurocognitive tests reflected slower reaction times and poorer visual and verbal memory than scores of TBI patients with headache only or no headache [9]. The same study also demonstrated higher scores during recovery from head injury, especially on tests sensitive to cognitive, emotional, sleep, and somatic problems, indicating a greater level of disability amongst migraineurs [9].

### 4.1. Diagnostic Migraine Screeners

#### 4.1.1. Nine-Item Screener-SA

All Nine-Item Screener items except Question 4 indicated that posttraumatic migraine sufferers experienced proportionately more symptoms than nonmigraine patients. These included pain severity, unilateral location, throbbing, nausea, symptoms of aura, photo- and phonosensitivity, and functional impairment. Question 9, which evaluates functional impairment due to headache during the last three months, was the strongest predictor of migraines. Furthermore, it demonstrated nearly as much sensitivity and specificity as the overall score. The present results therefore suggest that greater functional impairment may be the cardinal symptom that most distinguishes migraine patients from nonmigraine patients in the mild TBI population. Future studies could evaluate the efficacy of administering this question alone for more rapid diagnosis.

Migraine and nonmigraine groups did not differ on Question 4, indicating that the effect of physical exertion on migraine symptoms may not be as important as pain characteristics and autonomic effects.

The 9-Item Screener-SA differentiated migraine sufferers from nonmigraine sufferers on eight of nine items and demonstrated high sensitivity and specificity in an mTBI population. Indeed, it was comparable to the extensive physician diagnosis in the present study, which was based on several headache measures in addition to the Gold Standard ICHD-II diagnosis, family and personal medical history, and a neurological exam. This suggests that it could serve as a stand-alone diagnosis for classifying migraine headaches in an mTBI population, in both clinical and research designs. If the 9-Item Screener-SA is equally accurate at detecting migraine, this could reduce physician workload and improve the efficacy of healthcare delivery. However, these results must be interpreted with caution. More detailed physician examinations may lower the incidence of false positives that would threaten patient safety. For example, a patient presenting with migraine-type pain may require a more thorough consultation to differentiate migraine from a hematoma or cervical sprain. Misdiagnosing the latter as migraine is a significant safety risk that would also be costly as well to both the healthcare system and the patient.

#### 4.1.2. Three-Item ID Migraine Screener

Only light sensitivity differed between migraine patients and nonmigraine patients, with migraine sufferers experiencing greater sensitivity. Few studies have examined photosensitivity in TBI populations. Bohnen et al. suggested that photosensitivity in TBI patients may be due to inadequate inhibition of sensory processing by the orbitofrontal cortex in subcortical and posterior areas of the brain [19]. However, the present study suggests that migraine in a TBI population may play a more important role in photosensitivity than previously acknowledged. In cases where TBI does not directly injure the visual pathways, photosensitivity may not exist as a function of postconcussive syndrome. Rather, it may be a function of posttraumatic migraine. Migraine history before TBI is also believed to be a risk factor for developing light sensitivity after injury [20]. Interestingly, in our population, there were no significant differences in headache history between migraine patients and those without migraines.

Lastly, Lipton et al. determined that the Nine-Item Screener did not have any more sensitivity or specificity for detecting migraines in the general population than the 3-Item ID Migraine Screener [4]. In contrast, the present results indicate that the Nine-Item Screener inventory has the best positive predictive value and the best differentiation of migraine patients, even over that of the 3-Item ID Migraine Screener, when evaluating a mild TBI population.

### 4.2. Headache Impact Measure

#### 4.2.1. HIT-6

Similar to results on the Nine-Item Screener, the HIT-6 demonstrated that migraine patients suffered the greatest pain and functional limitations. Migraine and nonmigraine patients did not differ on questions three through six, which examine cognitive and emotional aspects such as concentration, fatigue, and irritability. One possible explanation is that these symptoms overlap with those of postconcussion syndrome, a common condition in the TBI population [21]. This is supported by the fact that there were no significant differences in groups on the RPQ.

Interestingly, the findings of the present study contrast with those of Kontos and colleagues, who demonstrated cognitive differences between PTH patients with and without migraines. This difference may have been due to the latter group's use of objective measures to investigate cognitive constructs that were more specifically defined [9]. We used self-report measures, which may have been more vulnerable to response changeability. Furthermore, their study focused exclusively on sports concussions, while ours included patients who had been in motor vehicle accidents, which are often associated with more complex traumas, and require more medications for conditions, such as pain, which may decrease cognitive ability independent of headaches. This could obscure subtle differences in cognitive function between migraine and nonmigraine groups.

### 4.3. Postconcussive Symptoms

#### 4.3.1. Rivermead Post-Concussive Symptoms Questionnaire (RPQ)

The lack of significant differences found between groups on the RPQ demonstrates that administering this questionnaire alone may result in failure to distinguish migraine headaches from other postconcussion symptoms in mTBI patients. The RPQ is an inventory of postconcussive symptoms, including headaches, irritability, difficulty in concentrating, phono- and photosensitivity, and nausea and vomiting. Previous studies have demonstrated that mTBI patients suffering from migraines often do not receive the treatments they require [8].

One limitation of the current study was the exclusion of asymptomatic patients who were seen in the emergency department without further referral to a clinic. The small sample size may also limit generalizability. In addition, among possible triggers for migraine, only motion sickness and food intolerance were examined. Other factors that trigger migraines could be explored in future studies. Finally, headaches were recalled by patients retrospectively. A daily headache log may have reduced any possible bias or memory deficits in patients' self-reports.

## 5. Conclusion

The present study demonstrated that the Nine-Item Screener may be the best inventory at detecting migraine headaches in a mild TBI population. Furthermore, the 9-Item Screener, when self-administered, appears to be equally accurate at identifying migraine patients as an extensive physician-administered diagnosis based on the 2004 ICHD-II criteria for migraine [16], the HIT-6, family history of headache, cervical sprain, childhood motion sickness, and food intolerance. However, the physician-administered assessment may still be instrumental in differentiating between migraines and other important types of headache. The current study also suggests that administering the RPQ alone may result in an oversight of the contribution of migraine headaches to postconcussion symptoms in mTBI patients.

Our work contributes to a sparse literature comparing the ability of different measures of headache to detect migraine in a TBI population. This is an important contribution, since migraine-specific disability may be related to protracted recovery from mild TBI, and, in the present study, items measuring pain severity, photosensitivity, and disability on the Nine-Item Screener, the HIT-6, and the 3-Item ID Migraine Screener may best differentiate migraine sufferers from nonmigraine sufferers. It is not yet known whether it is appropriate to generalize these results to all mTBI patients with migraine. However, the findings from this study could contribute to a more standardized approach to classifying migraine headaches in a TBI population, for the optimization of clinical and research designs. Further research is necessary to better classify posttraumatic headache types and conduct more effective research on the contribution of migraine to disability. Migraine-targeted treatments could help increase functional outcome and reduce healthcare costs related to protracted recovery times from traumatic brain injury.

## Figures and Tables

**Table 1:** Demographics and previous medical history.

Variable	
Total number of patients	43
Age at injury (M ± SD)	44.8 ± 18.3
Male	48.8
Female	51.2
Medical history	
History	
Previous migraines	
No	31 (79%)
Yes	8 (21%)
Chronic headaches	
No	21 (55%)
Yes	17 (45%)
Motion sickness	
No	25 (86%)
Yes	4 (14%)
Food intolerance	
No	26 (90%)
Yes	3 (10%)
ICHD-II diagnosis	
Migraines	
No	23 (53%)
Yes	20 (47%)
Accident variables	
TBI etiology	
Fall	16 (38%)
MVA	8 (19%)
MVA (pedestrian or cyclist)	3 (7%)
Assault	3 (7%)
Suicide attempt	3 (7%)
Sports	8 (19%)
Other	1 (2%)
LOC	
No	22 (59%)
Yes	15 (41%)
Duration PTA	
None	14 (38%)
<5 min	3 (8%)
5–10 min	3 (8%)
10–15 min	2 (5%)
15–30 min	2 (5%)
30–60 min	5 (14%)
60+ min	8 (22%)

**Table 2:** Comparison of headache symptoms in migraine and no migraine groups on the Nine-Item Screener.

Item	Overall (%)	Migraine (%)	No migraine (%)	Chi-square test	*p* value
Q1^*∗∗*^	48.8	70.0	30.4	6.70	.010
Q2^*∗∗*^	67.4	90.0	47.8	8.67	.003
Q3^*∗∗*^	81.4	100	65.2	8.55	.003
Q4	69.8	80.0	60.9	1.86	.173
Q5^*∗*^	42.9	60.0	27.3	4.58	.032
Q6^*∗∗*^	44.2	70.0	21.7	10.10	.001
Q7^*∗∗*^	76.7	95.0	60.9	6.98	.008
Q8^*∗∗*^	83.7	100	69.6	7.27	.008
Q9^*∗∗∗*^	62.8	95.0	34.8	16.60	<.001

Chi-square tests demonstrated that migraine patients had significantly more symptoms and lower functioning than the no migraine group on all questions except number four.

^*∗*^ *p* < .05.

^*∗∗*^ *p* < .01.

^*∗∗∗*^ *p* < .001.

**Table 3:** Sensitivity and specificity of the Nine-Item Screener in patients with and without migraine.

Item	Sensitivity (%)	Specificity (%)	95% CI AUC
(1) Pain is worse on just one side	70	69.6	0.54–0.83
(2) Pain is pulsing, pounding, or throbbing	90	52.2	0.56–0.85
(3) Pain is moderate or severe	100	34.8	0.52–0.81
(4) Pain is made worse by activities such as walking or climbing stairs	80	39.1	0.44–0.75
(5) You feel nauseated or sick to your stomach	60	72.7	0.50–0.80
(6) You see spots, stars, zigzag lines, or gray area for several minutes or more before or during your headaches	70	78.3	0.59–0.87
(7) Light bothers you (a lot more than when you do not have headaches)	95	39.1	0.52–0.81
(8) Sound bothers you (a lot more than when you do not have headaches)	100	30.4	0.49–0.79
(9) Functional impairment due to headache in the last three months	95	65.2	0.64–0.90

Note: *N* = 43.

**Table 4:** Comparison of headache impact symptoms in migraine and no migraine groups on the HIT-6.

Item	Chi-square test	*p* value (%)
(1)^*∗*^ When you have headaches, how often is the pain severe?	11.47	.022
(2)^*∗*^ How often do headaches limit your ability to do usual daily activities including household work, work, school, or social activities?	9.57	.048
(3) When you have a headache, how often do you wish you could lie down?	2.51	.642
(4) In the past 4 weeks, how often have you felt too tired to do work or daily activities because of your headaches?	6.02	.198
(5) In the past 4 weeks, how often have you felt fed up or irritated because of your headaches?	8.04	.090
(6) In the past 4 weeks, how often did headaches limit your ability to concentrate on work or daily activities?	5.01	.286

Chi-square tests demonstrated that migraine patients had significantly more symptoms and lower functioning than the no migraine group on questions 1 and 2. There were no significant differences between groups on any other questions.

^*∗*^ *p* < .05.

**Table 5:** Sensitivity and specificity of the 3-Item ID Migraine Screener in patients with and without migraine.

Item	Sensitivity (%)	Specificity (%)	95% CI AUC
Limiting activities	79.0	36.4	0.42–0.74
Talking to physician	73.7	33.3	0.36–0.69
Lipton 1: nausea	63.2	45.5	0.37–0.69
Lipton 2: light sensitivity	84.2	54.6	0.52–0.82
Lipton 3: limited for ≥ one day	84.2	40.9	0.47–0.78

Note: *N* = 41.
